# The impact of travelling on the COVID-19 infection cases in Germany

**DOI:** 10.1186/s12879-022-07396-1

**Published:** 2022-05-12

**Authors:** Moritz Schäfer, Karunia Putra Wijaya, Robert Rockenfeller, Thomas Götz

**Affiliations:** Mathematical Institute, University of Koblenz–Landau, 56070 Koblenz, Germany

**Keywords:** COVID-19, Epidemiology, Disease dynamics, Travellers, SEIRD-model, Parameter estimation, Metropolis algorithm, BIC, Sensitivity analysis, Reproduction number

## Abstract

**Background:**

COVID-19 continues to disrupt social lives and the economy of many countries and challenges their healthcare capacities. Looking back at the situation in Germany in 2020, the number of cases increased exponentially in early March. Social restrictions were imposed by closing e.g. schools, shops, cafés and restaurants, as well as borders for travellers. This reaped success as the infection rate descended significantly in early April. In mid July, however, the numbers started to rise again. Of particular reasons was that from mid June onwards, the travel ban has widely been cancelled or at least loosened. We aim to measure the impact of travellers on the overall infection dynamics for the case of (relatively) few infectives and no vaccinations available. We also want to analyse under which conditions political travelling measures are relevant, in particular in comparison to local measures. By travel restrictions in our model we mean all possible measures that equally reduce the possibility of infected returnees to further spread the disease in Germany, e.g. travel bans, lockdown, post-arrival tests and quarantines.

**Methods:**

To analyse the impact of travellers, we present three variants of an susceptible–exposed–infected–recovered–deceased model to describe disease dynamics in Germany. Epidemiological parameters such as transmission rate, lethality, and detection rate of infected individuals are incorporated. We compare a model without inclusion of travellers and two models with a rate measuring the impact of travellers incorporating incidence data from the Johns Hopkins University. Parameter estimation was performed with the aid of the Monte–Carlo-based Metropolis algorithm. All models are compared in terms of validity and simplicity. Further, we perform sensitivity analyses of the model to observe on which of the model parameters show the largest influence the results. In particular, we compare local and international travelling measures and identify regions in which one of these shows larger relevance than the other.

**Results:**

In the comparison of the three models, both models with the traveller impact rate yield significantly better results than the model without this rate. The model including a piecewise constant travel impact rate yields the best results in the sense of maximal likelihood and minimal Bayesian Information Criterion. We synthesize from model simulations and analyses that travellers had a strong impact on the overall infection cases in the considered time interval. By a comparison of the reproductive ratios of the models under traveller/no-traveller scenarios, we found that higher traveller numbers likely induce higher transmission rates and infection cases even in the further course, which is one possible explanation to the start of the second wave in Germany as of autumn 2020. The sensitivity analyses show that the travelling parameter, among others, shows a larger impact on the results. We also found that the relevance of travel measures depends on the value of the transmission parameter: In domains with a lower transmission parameter, caused either by the current variant or local measures, it is found that handling the travel parameters is more relevant than those with lower value of the transmission.

**Conclusions:**

We conclude that travellers is an important factor in controlling infection cases during pandemics. Depending on the current situation, travel restrictions can be part of a policy to reduce infection numbers, especially when case numbers and transmission rate are low. The results of the sensitivity analyses also show that travel measures are more effective when the local transmission is already reduced, so a combination of those two appears to be optimal. In any case, supervision of the influence of travellers should always be undertaken, as another pandemic or wave can happen in the upcoming years and vaccinations and basic hygiene rules alone might not be able to prevent further infection waves.

## Introduction

### Background

**Fig. 1:**
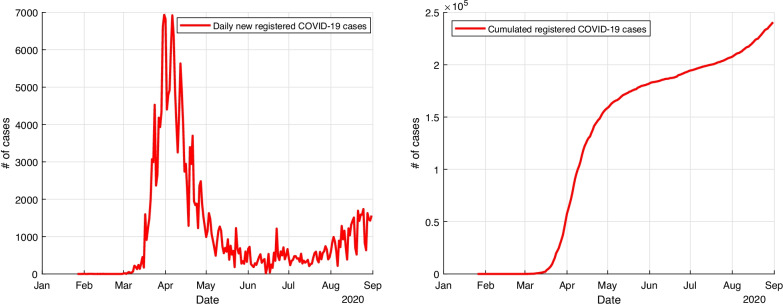
Daily confimed cases (left) and cumulative confirmed cases (right) with COVID-19 in Germany from January 26 until August 31, 2020 according to Johns Hopkins University

The COVID-19 disease in Germany started with a first infection case on 26 January 2020 in Bavaria [1]. In March, the number of cases grew rapidly (with a maximum of 6933 cases on 27 March), and various social restrictions were imposed as an active intervention of the disease [2, 3]. On June 10, only 16 new infection cases were detected [2]. In mid June, travel related restrictions were relaxed within Europe [4]. However, the pandemic continued to spread worldwide and by the end of August, new maxima for the daily cases worldwide set another record for that time [5]. Towards the end of the summer holidays in the first German states in mid to end of August, a second rise of incidence happened with over 1000 new infection cases per day [2]. Figure 1 shows the temporal evolution of COVID-19 cases in Germany from 26 January until 31 August, as reported by the Johns-Hopkins-University (JHU). The daily registered COVID-19 are shown on the left side; on the right side, the cumulative registered cases can be seen.

According to the Robert Koch Institute (RKI), a governmental institute for disease control in Germany, many of the cases from June onwards were directly related to German travellers returning home from abroad [6]. Given already long implementation of such travel restrictions (as of 2022), studies that evaluate their effectiveness in Germany are limited. Internationally, however, relevant studies have been preceding and may provide insights for ensuing ones. Siegenfeld et al. [7] propose and estimate a region-to-region reproduction number, as opposed to the usual person-to-person reproduction number, by assuming that the number of other regions infected by a ‘central’ region follows a Poisson process. The number appears to be linearly dependent on the probabilities of an infected individual from the central region to travel outside the region, before and after the imposition of travel restrictions. Accordingly, supervision of the number of travellers becomes one of the decisive parameters to contain the spread of COVID-19. They conclude that if high-risk areas impose travel measures coupled with social measures shortly after community transmission, then the reduction of travellers becomes the determining factor if the outbreak can be eliminated. However, without timely social measures that manage to reduce the local reproduction number to a value below 1, travel restrictions only lead to a delay in the spread of epidemics. Chinazzi et al. [8] emphasize a more broad-minded definition of travel restrictions to include case detection and behavioral changes, as the lone flight traffic limitations around Wuhan in January 2020 (up to 90%) could have only returned a modest containment effect. However they found that, while initially effective—as case importations were reduced by nearly 80% until mid-February by international travel restrictions after 2–3 weeks the effect was reduced and numbers grew outside China. Zou et al. [9] introduced a multi-patch transportation model and also studied the effects of vaccination and quarantine on the disease dynamics of such a multi-patch model. As a result they propose to control travel or migration in high-risk areas while interventions in low-risk areas are less effective. Leung et al. [10] also address the combination of traffic flow reduction and testing–quarantine for inbound travellers toward reopening the economy as a good response in case of weakening public health and social measures (PHSMs) and vaccine limitation. A systematic review over the impact of travel restrictions on influenza can be found in Mateus et al. [11]. The WHO review considers the effectiveness of internal and external travel restrictions and concludes only very strict restrictions would be expected to have an impact on influenza transmission, but the evidence on these results is proclaimed as low. It is also stated that extensive travel restrictions cause meaningful reduction of the spread of influenza, but only in terms of a delay of several weeks or months, not in terms of containing the disease in areas of high risk. Also, correspondence by Hollingsworth et al. [12] conveys that for scenarios with few infection cases and a low reproductive value $\mathcal R$, travelling can help to contain diseases, while for a higher value of $\mathcal R$ only very hard travelling restrictions can prevent spread or postpone the possible wave to a later date, so they conclude that given those latter circumstances, country-based transmission reduction is to be preferred over travel restrictions. Epstein et al. [13] used stochastic epidemic models to explore the role of international (air) travel restrictions, and also found that strong interventions in travelling can lead to a short-time delay in the spread of epidemics, but they state that this ‘saved’ time can be effectively used by other disease control measures. Another systematic review by Grépin et al. [14] regarding the effectiveness of travel restrictions has compared the results to the role of travel restrictions on influenza. The authors find that the recommendations of WHO [15] do not necessarily apply to those of COVID-19 as it remains unclear if the findings on influenza can be compared here. Travel measures implemented in Wuhan are found to be effective at the reduction of cases both nationally and internationally, and are more effective when those measures are undertaken early (i.e. in the outbreak). A diffusion-based and non-international approach can be found in Berestycki et al. [16]. The authors find that fast diffusion effects along major roads are an important factor of the spread of epidemics like COVID-19 in Italy and HIV in the Democratic Republic of Congo.

### Structure of the paper

In the present study our first question is the following:

(Q0) How can we model the spread of infections in Germany with inclusion of travellers to measure the impact they have on the overall numbers?

A susceptible–exposed–infected–recovered–deceased (SEIRD) model introduced in the previous work of Heidrich et al. [17] is used as the foundation for any of the applied systems. In the most simple version, we use a system which does not include travellers as a reference. As a next step, we set up another SEIRD model for Germany which includes travellers to the respective countries and estimate both the ‘classical’ parameters and also the impact of the infected travellers to the overall epidemics. In one variation of the model, the impact of travellers is assumed to be constant over time, while in a second formulation, we allow a time-dependent value as awareness of the population and political policies might change over time. The travellers are assumed to be part of the infection cycle in the respective destination countries, for which we have also set up another (aiding) SEIRD model. We estimate the relevant model parameters by using the available data from the Johns-Hopkins University (JHU) [2]. The estimation of several disease-related parameters like e.g. the transmission rate, death rate or detection rate as of Heidrich et al. [17] is based on a least-squares fit between the model output and the reported data, where both the reported infections and fatalities are taken into account. Furthermore, the three models are compared in terms of validity and simplicity. Posterior to the fitting and parameter estimation, two questions remain in discussions:

1. Which variable parameters need more careful specifications for which the model solutions, as well as the likelihood function, easily perturb within large orders of magnitude as these parameters slightly change?
2. Which interventions (local interventions like social distancing, masks, lockdown etc. or travelling restrictions) should be more emphasized for possible variations of parameter values in the prediction window?

Our goal will then be to answer these questions with the help of two measures: At first, we consider a time-dependent measure, in which we observe which parameters have a larger impact on the outcome of the five (S,E,I,R,D) compartments. Further, we consider time-independent measures and compare local measures (identified by the overal transmission rate) and travel measures (identified with the newly introduced travel impact rate) and regard under which circumstances one or the other are more relevant for the infection cases. Using the results of questions (Q0), (Q1) and (Q2), we aim to give an answer on the relevance of travel restrictions on the Corona or other infectious diseases in general, and to investigate under which conditions travel restrictions can be a more powerful tool than other non-pharmaceutical measures.

## Methods

All methods were performed in accordance with the relevant guidelines and regulations.

### Data

The incidence data used in this study are daily registered COVID-19 cases and deceased cases from Germany (see Fig. 1) and other countries from 1 June until 31 August, 2020. Due to the usual independent and identically distributed (iid) assumption on the measurement error (cf. King et al. [18]), only the daily incidence data will be used for optimization parameter estimation of the later introduced models. To accompany the modeling, population data from all countries in consideration are taken from UN data [19]. We only include European countries with available traveller statistics and countries outside of Europe with a total sum of more than 5000 travellers in the travelling statistics. For the close European countries, the number of travellers is estimated by the travel statistics of 2020 for German travellers [20] (for relative shares) and hospitality statistics in Germany for foreign travellers [21]; these numbers are generally subtracted from the total amount of flight passengers. The number of travellers from and to farther and non-European countries is gained from analysis of the flight passengers from the respective country [22]. In some larger countries, namely USA, Russia, China, and Japan, the data was problematic. Flight routes from and to these countries are often non-direct, so the plain values of flight passengers would underestimate the real amount of travellers to these countries. As a compromise, we assumed the amount of German travellers to those countries to be the same as the number of foreign visitors from those countries in Germany, which makes this estimation more meaningful. The populations and amount of travellers per month of this total of 55 countries is presented in Table 2.

### Model

#### Traveller induced model

In the previous work we investigated the dynamics of COVID-19 disease until early May 2020 [17]; this study departs from this approach. Again we use a variation of the SIR-model introduced by Kermack and McKendrick [23]; see also Martcheva [24] for an overview of mathematical models in epidemiology. It builds up on delayed differential equation (DDE) system to describe the behaviour of the disease in Germany in summer 2020. While the use of stochastic variables can make the model more realistic, but may also lead to further technical questions including noise type, stable ergodicity, and predictability, which go beyond the original scope. Therefore, we tested a deterministic model for the main aim. The entire population *N* is subdivided into five compartments: susceptible *S*, exposed *E*, infected *I*, recovered *R*, and deceased *D*, so that we deal with a so-called SEIRD model. The virus is transmitted from infected persons to susceptible persons at a piecewise constant rate $\beta$. After an incubation duration $\kappa ^{-1}$ exposed individuals become infective. Loss of infectivity is gained after an average duration $\gamma ^{-1}$; the death parameter $\mu$ describes the probability for infected persons dying from the disease. A time lag $\tau$ between the infected and the deceased state accounts for the fact that the number of people dying from the disease is attained from the infected number $\tau$ days earlier. Here, we also introduce an additional compartment: travellers $E_t$ which have been exposed to the disease abroad. Values for the fixed model parameters in Germany are given in Table 1.

**Table 1:** Used parameter values

Parameter	Value	References
*N*	83,019,213	[19]
$$\kappa$$	(3 d$$)^{-1}$$	[25]
$$\gamma$$	(10 d$$)^{-1}$$	[25]

These assumptions lead us to the following five-dimensional ODE system.

1a $$\begin{aligned} &\dot{S}= - \frac{\beta }{N} \,S \,I - E_T(t) \\ &S(t_0)=S_0=N - E_0 - I_0 - R_0 - D_0>0 \end{aligned}$$

1b $$\begin{aligned} \dot{E}= \frac{\beta }{N} \,S\, I + E_T(t) - \kappa \, E \qquad E(t_0)=E_0\ge 0 \end{aligned}$$

1c $$\begin{aligned} & \dot{I}= \kappa \, E - \gamma \,\big ( (1-\mu )\,I +\mu \,I(t-\tau ) \big )\\&I(t_0-\tau \le t\le t_0) = \varphi (t)>0 \end{aligned}$$

1d $$\begin{aligned} \dot{R}= \left( 1 - \mu \right) \gamma \,I \qquad R(t_0)=R_0 \ge 0 \end{aligned}$$

1e $$\begin{aligned} \dot{D}= \mu \, \gamma \,I(t-\tau ) \qquad D(t_0)=D_0 \ge 0 \end{aligned}$$

Let $X=(X_i)$ and $Z=(Z_i)$ denote the daily new confirmed cases and deaths related to COVID-19 in Germany. The subscript *i* serves to point out the measurement at time point $t_i$ as reported by the JHU [2]. Not all infections are by nature detected, from which case we introduce detection rates $\delta$ for Germany and $\delta _j$ for the destination country, respectively. For the persons which are currently infected or have recovered, we assume that only this proportion $\delta$ or $\delta _j$ is tested and detected and hence appears in the statistics; however, we assume no undetected deceased cases. We assume that the proportion of detected cases versus real infections is constant over the whole time interval, so that no temporal change of the detection rate is needed in our model. The initial value of the infected cases at the starting date $t_0$ is later on subject of the estimation procedure. Therefore, we use the infected data as the real data divided by the detection rate, for Germany and destination countries, respectively:

2 $$\begin{aligned} \varphi (t):= \frac{\text {interp}\{(X_i)\}(t)}{\delta } \qquad t_0-\tau \le t\le t_0. \end{aligned}$$

As travel measures are relaxed as of June 15, we designed the starting time $t_0$ of this model as June 1. This way we allow parameter estimation of the transmission rate $\beta$ in the first two weeks which is fully independent of the travel impact rate $\alpha$, so those parameters are not correlated during the optimization process (note that in Eq. (3) those parameters are multiplied with each other). The end date is fixed to 31 August because of the end of summer holidays (in most German states) and new restrictions in other countries from September onwards, e.g. a travel warning for Spain [4], which will affect the transmission parameters. The initial values are either gained from the JHU data sets [2] or introduced as free parameters which have to be optimized in the Metropolis algorithm. The function $\varphi :[t_0-\tau , t_0]\rightarrow {\mathbb {R}}_+$ denotes the initial history of the infected required for the well-posedness of the above DDE; the value $\tau$ is another free parameter. The number of travellers which have been exposed to the disease is defined as

3 $$\begin{aligned} E_T(t)= \alpha (t)\, \sum _j \frac{\beta ^{(j)}(t)}{N^{(j)}} \, { T_{\text {(0)}\leftrightarrow (j)}(t)\, I^{(j)}(t)}. \end{aligned}$$

The values $I^{(j)}$ and $N^{(j)}$ are defined by the number of infected people and respectively the resident population in country $(j)\ne (0)$ at time *t*. The function $T_{\text {(0)}\leftrightarrow (j)}(t)$ describes the number of travellers from Germany to country *j*, whereby the superscript (0) denotes Germany from now on. Travellers are assumed to have a higher risk of getting infected, due to being more active, visiting places and travelling (e.g., in a plane) with more contacts than an average resident. Therefore, we define $\alpha (t)$ to quantify the special risk of getting infected as a traveller. If $\alpha \equiv 1$, then the transmission rate for travellers is equal to the country’s specific transmission rate $\beta ^{(j)}(t)$. This rate is piecewise constant with switching returned from imposition or relaxation of certain measures. No inclusion of travellers due to bans or closed borders are identical to $\alpha \equiv 0$.

#### Infection rate induced model

As we aim to estimate $\beta _j(t)$ and $I^{(j)}$ for all relevant countries, we have to set up another ODE system modelling the disease dynamics. Let (*j*) therefore be the specific country. For all countries $(j), j\in \{1,2,\dots ,M-1,M\}$ with *M* being the amount of observed countries, we estimate the local transmission rate $\beta _j(t)$ as well as the amount of infected persons $I^{(j)}(t)$ for all relevant time points by using an SEIRD model without a traveller compartment, while the total population $N^{(j)}$ is assumed to be constant over time.

4a $$\begin{aligned} & \dot{S}^{(j)}= - \frac{\beta _j(t)}{N^{(j)}} \,S^{(j)}\, I^{(j)} \\ & S^{(j)}(t_0)=S^{(j)}_{0}=N^{(j)} - E^{(j)}_0 - I^{(j)}_0 - R^{(j)}_0 - D^{(j)}_0>0 \end{aligned}$$

4b $$\begin{aligned} \dot{E}^{(j)}= \frac{\beta _j(t)}{N^{(j)}}\, S^{(j)} \,I^{(j)} - \kappa \quad E^{(j)} \dot{E}^{(j)}(t_0)=E^{(j)}_0\ge 0 \end{aligned}$$

4c $$\begin{aligned} &\dot{I}^{(j)}= \kappa \, E^{(j)} - \gamma \,\big ( (1-\mu _j)\,I^{(j)} +\mu _j\, I^{(j)}(t-\tau _j) \big ) \\ & I^{(j)}(t\le t_0) = \varphi ^{(j)}(t) >0 \end{aligned}$$

4d $$\begin{aligned} \dot{R}^{(j)}= \left( 1 - \mu _j\right) \,\gamma \,I^{(j)} \qquad R^{(j)}(t_0)= R^{(j)}_0 \ge 0 \end{aligned}$$

4e $$\begin{aligned} \dot{D}^{(j)}= \mu _j\, \gamma \,I^{(j)}(t-\tau _j) \qquad D^{(j)}(t_0)=D^{(j)}_0 \ge 0 \end{aligned}$$

Let again $X^{(j)}=(X^{(j)}_i)$ and $Z^{(j)}=(Z^{(j)}_i)$ denote the daily infection and death cases in the respective destination country as reported by the JHU [2]. Then, the history function is denoted analogously to before by

5 $$\begin{aligned} \varphi _j(t)&:= \frac{\text {interp}\{(X^{(j)}_i)\}(t)}{\delta _j}\qquad&t_0-\tau _j\le t\le t_0. \end{aligned}$$

The values for $\kappa$ and $\gamma$ are assumed to be independent of country (*j*). In the datasets for the countries, we find a sudden ‘step’ in the infection rates. This can not be modelled by travellers like in the model for Germany, which has two reasons: (1) Traveller data is not available for each country. (2) The reasons for the raised infection numbers in other countries are not of interest for the traveller model in Germany. Instead of using an additional parameter $\alpha$ and a traveller compartment, we assume the transmission rates to be piecewise constant. By performing various simulations, the best-fitting ‘switching date’ where the rate is allowed to change value is found to be 20 July:

6 $$\begin{aligned} \beta _j(t) := {\left\{ \begin{array}{ll} \beta ^{(j)}_0, &{} t \le 19 \text { July} \\ \beta ^{(j)}_1, &{} 20 \text { July} \le t \\ \end{array}\right. } \end{aligned}$$

This system (4) is used both for the destination countries of German travellers and also for the model for Germany which does not include travellers (later on to be called model A). In the latter case, we can see the system as a special case of system (1) with $j=0$, representing Germany. Travel restrictions are being relaxed as of 15 June [26]. This date is therefore assigned to be the starting time $t_0$ for the destination countries, while the starting date remains 1 June for the no-travel model for Germany. The end date remains 31 August (in both cases) as we require the values of $\beta _j$ and $I^{(j)}$ until the end of the observed time interval, and of course nothing changes for the German model. The parameters $N^{(j)}$ reflect the current total populations in all regarded countries which are the destination or origin of travellers from and to Germany; the population values are taken from UN data [19]. Results using the optimized parameters are also shown in Table 2.

**Table 2:** Fixed parameter values for the population $N_j$ as well as the (estimated) number of Germans travelling to the respective country *j*, namely $T_{\text {Germany}\leftrightarrow j}$, per month in summer 2020 and the transmission parameters $\beta _{j,1/2}$ by application of Eq. (4)

Country	Population	Travellers			Transmission	
		June	July	August	$\beta _{j,1}$	$\beta _{j,2}$
Decimal power/unit	10$^6$	1	1	1	10$^{-1} d^{-1}$	10$^{-1}d^{-1}$
Albania	2.88	945	3366	9505	1.20	1.19
Austria	8.90	312,364	636,414	782,818	1.35	1.36
Belarus	9.45	1595	1985	3102	0.38	0.65
Belgium	11.51	36,210	155,295	92,495	1.17	1.60
Bosnia and Herzegovina	3.30	2811	2849	6702	1.54	1.10
Brazil	211.05	6715	4366	3778	1.22	1.12
Bulgaria	6.95	11,562	42,552	74,363	1.24	1.06
Canada	37.41	4746	9778	8368	0.31	1.33
China	1433.78	3711	5921	7077	1.55	1.05
Croatia	4.06	66,029	84,952	150,790	0.31	1.10
Cyprus	0.89	360	7191	14,049	0.42	1.89
Czech Republic	10.69	51,518	130,651	148,353	0.84	1.41
Denmark	5.81	48,986	395,924	571,649	0.77	1.70
Egypt	100.39	2542	5134	7790	0.65	0.36
Estonia	1.33	1006	3380	5967	0.23	2.00
Ethiopia	112.08	1431	2089	2066	1.84	1.45
Finland	5.53	4624	12,134	19,074	0.31	1.74
France	67.20	105,905	326,298	345,913	0.95	1.96
Greece	10.7	15,930	179,531	372,892	1.41	1.75
Hungary	9.77	30,154	53,080	71,577	0.36	1.71
Iceland	0.34	889	7892	13,718	1.66	1.56
Ireland	4.97	4892	8965	9065	0.43	2.20
India	1366.42	5168	8676	14,046	1.39	1.25
Israel	88.52	2455	2693	797	1.65	1.23
Italy	60.29	126,855	272,324	415,581	0.21	1.75
Japan	126.86	1457	2340	3292	1.78	1.29
Kosovo	1.72	586	7341	18,626	1.59	1.10
Latvia	1.91	5936	12,637	20,798	0.31	1.52
Lebanon	6.87	167	1699	5298	1.09	1.94
Lithuania	2.79	1203	1787	2415	0.49	1.89
Luxembourg	0.63	4562	4466	2946	2.54	0.51
Malta	0.51	261	9338	16,974	1.16	1.95
Mexico	127.58	2079	2726	2253	1.70	0.69
Montenegro	0.63	728	2490	4118	3.75	0.54
Moldova	4.04	972	1728	3815	0.85	1.30
Netherlands	17.40	188,840	721,721	1,592,831	1.04	1.72
Northern Macedonia	2.08	0	3486	9875	0.89	1.02
Norway	5.37	8326	42,589	64,125	0.74	1.70
Poland	27.94	95,372	171,127	268,559	0.52	1.51
Portugal	10.29	17,659	63,369	111,867	0.51	0.81
Qatar	2.83	6063	8336	6747	0.79	1.00
Romania	19.36	5702	32,822	41,255	1.18	1.35
Russia	145.87	3550	6017	7324	0.61	1.03
Serbia	8.77	5164	5577	9672	1.62	0.61
Slovakia	5.46	19,372	31,161	56,401	1.16	1.54
Slovenia	2.07	3759	5361	5987	1.44	1.14
Spain	47.32	22,209	331,894	436,624	1.19	1.86
Sweden	10.32	9050	39,584	46,878	0.38	0.55
Switzerland	8.50	102,698	272,121	388,971	1.49	1.35
Tunisia	11.69	644	2709	11,292	1.09	2.12
Turkey	83.43	36,986	144,350	343,972	0.77	1.14
United Kingdom	66.43	17,026	29,925	32,969	0.92	1.16
Ukraine	43.99	3020	8934	14,759	0.77	1.44
United States of America	329.06	24,123	42,409	41,613	0.81	1.62
United Arab Emirates	9.77	3231	9394	6856	0.59	1.10

#### Travellers and travel impact rate

We only include European countries with available traveller statistics and countries outside of Europe with a total sum of more than 5000 travellers in the travelling statistics. For the close European countries, the number of travellers is estimated by the travel statistics of 2019 and 2020 for German travellers [20] (for relative shares) and hospitality statistics in Germany for foreign travellers [21]. The number of travellers from and to farther and non-European countries is gained from analysis of the flight passengers from the respective country [22]. In some larger countries, namely USA, Russia, China, and Japan, the data was problematic. Flight routes from and to these countries are often non-direct, so the plain values of flight passengers would underestimate the real amount of travellers to these countries. As a compromise, we assumed the amount of German travellers to those countries to be the same as the number of foreign visitors from those countries in Germany, which makes this estimation more meaningful. The populations and amount of travellers per month of this total of $M=55$ countries is presented in Table 2.

By using this table, we can compute the daily value for $T_{\text {(0)}\leftrightarrow (j)}$ by the number of travellers divided by the days in the respective month. E.g., for June, only the 16 days from 15 June to 30 June are considered. Average time of spending time here is 12 days so e.g. for July, we have $T_{(0)\leftrightarrow (j)}=$ 331,894 $\cdot \frac{12}{31\text {d}} \approx$ 128,475 day$^{-1}$. The uncertainty in the value of 12 days for the average travel length is mitigated by the estimation of $\alpha$, as these two values are directly multiplied and thus only the product of those two values is important.

In model B, $\alpha (t)$ is assumed to be constant over time as soon as the travel ban is loosened:

7 $$\begin{aligned} \alpha (t) := {\left\{ \begin{array}{ll} 0 &{}t \le \text {14 June} \\ \alpha &{} 15 \text { June} \le t \le 31 \text { August} \\ \end{array}\right. } \end{aligned}$$

In model C, we define a piecewise constant function $\alpha (t)$ as follows:

8 $$\begin{aligned} \alpha (t) := {\left\{ \begin{array}{ll} 0 &{}t \le \text {14 June} \\ \alpha _{0} &{} 15 \text { June} \le t \le 30 \text { June} \\ \alpha _{1} &{} 1 \text { July} \le t \le 31 \text { July} \\ \alpha _{2} &{} 1 \text { August} \le t \le 31 \text { August} \\ \end{array}\right. } \end{aligned}$$

This way, we are able to identify temporal differences in the travelling compartment, e.g. caused by a different social behaviour or loosened restrictions. The last three ‘switching points’ are arbitrarily chosen at the beginning of each month to account for the time-dependency of $\alpha$.

### Models, parameter bounds and initial values

The parameters to be estimated in Eqs. (1) and (4) are transmission rate, detection rate, lethality, time lag, travel impact rate and numbers of exposed on 1 June 2020 (Germany) respectively 15 June 2020 (all other countries). The optimal parameters $u^{(j)*}$ and $u^*$ are determined by solving the following maximization problems in the respective models. This results in consideration of the following three models, with an auxiliary model being pre-evaluated before handling models B and C.

**Model A**: Time-dependent transmission rate, starting 1 June

9 $$\begin{aligned}&\max _{u^{(0)}}\, L(u^{(0)}) \qquad \text {subject to ODE (4)} \\ \text {where}\quad &u^{(0)} = \left( \beta ^{(0)}_0,\beta ^{(0)}_1,\delta _0,\mu _0,\tau _0,E^{(0)}_0\right) \in {\mathbb {R}}^{6} \end{aligned}$$

**Auxiliary model for models B and C**: For all countries $j=1,\dots , 55$, starting 15 June

10 $$\begin{aligned}&\max _{u^{(j)}}\, L(u^{(j)}) \qquad \text {subject to ODE (4)} \\ \text {where}\quad &u^{(j)} = \left( \beta ^{(j)}_0,\beta ^{(j)}_1,\delta _j,\mu _j,\tau _j,E^{(j)}_0\right) \in {\mathbb {R}}^{6}&\end{aligned}$$

**Model B**: Constant travel transmission parameter $\alpha (t)$ from 15 June onwards

11 $$\begin{aligned}&\max _{u}\, L(u) \qquad \text {subject to ODE (1)} \\ \text {where}\quad &u = \left( \beta ,\delta ,\mu ,\tau , \alpha ,E_0\right) \in {\mathbb {R}}^{6} \end{aligned}$$

**Model C**: Piecewise linear travel transmission function $\alpha (t)$ starting 15 June and jumps on 1 July and 1 August

12 $$\begin{aligned} &\max _{u}\, L(u) \qquad\, \text {subject to ODE (1)} \\ \text {where}\quad &u = \left( \beta ,\delta ,\mu ,\tau , \alpha _0, \alpha _1, \alpha _2,E_0\right) \in {\mathbb {R}}^{8} \end{aligned}$$

Table 3 shows the constraints for all parameters in the three models, which can also be used for $u_j$ (with the starting values $R_0$ and $Z_0=D_0$ as listed on the JHU website [2]).

**Table 3:** Parameter constraints with the respective constraints of the fitted parameters

$\beta _{0/1}$	$\delta$	$\mu$	$\tau$	$\alpha _j$	$N_0$	$E_0$	$I_0$	$R_0$	$D_0$
$> 0.05$	$0.05{-}1$	$\le 0.1$	$3{-}40$	$>0$	82,846,340	$> 0$	9,$407/\delta$	165,$632/\delta$	8555

Previous investigations by Götz and Heidrich [27] and Heidrich et al. [17] already give us orders of magnitude for the initial values of the optimization for $\beta _i$ and $\delta$. The order of magnitude of the time interval between the onset of infectiousness and death is derived from RKI modelling studies [25]. We allow a larger span in $\tau$ and $\tau _j$ than in [17] because the onset between infection and death is also dependent of the date on which the death case is registered in the statistics, where significantly different values depending on the country are possible here. A potential reason for this lies in different policies and procedures in reporting infection and death cases. The starting values at time $t_0$ for the detected cumulated infected $X_0$, detected recovered $Y_0=\delta R_0$ and detected dead $Z_0$ can be taken from the statistics. The initial number of infected is then defined as $I_0=(X_0-Y_0-Z_0)/\delta$. Depending on the detection rate $\delta$, the ‘real’ numbers $I_0$ and $R_0$ can be calculated by dividing those detected values by $\delta$. For the initial guess on the ‘real’ number of exposed individuals $E_0$ at time $t_0$, we use a derivation using the Basic Reproduction Number ${\mathscr {R}}_0$, which indicates how many new infections an infected individual causes on average during its illness in an otherwise susceptible population. In our model, the share of infected persons $I_0$ can either be at the start, the middle or the end of the infection, so several possible time stages of the infections are possible. The middle of this time interval is assumed to be the mean of all infected persons at time $t_0$. Thus, up to this point in time they could infect about ${\mathscr {R}}_0/2\cdot I_0$ persons on average which then become exposed to the virus, i.e. this is identical to $E_0$. Here, we assume that the initial basic reproductive number is approximately ${{\mathscr {R}}}_0\approx 1$ because of the stagnation of cases on a low level at the beginning of June.

### Likelihood function

As seen in the previous section, the unknown parameter sets $u^{(j)}$ and *u* will be estimated by maximisation of a likelihood function, which will be developed in this section. Note that the derivation of the function is described in detail only for *u*, but is equivalent for the likelihood function of $u^{(j)}$.

We denote ${{\tilde{I}}}$ and ${{\tilde{R}}}$ as the difference between the daily infection cases, i.e. for $i=1 \dots N$:

13 $$\begin{aligned} {{\tilde{I}}}_i&= \{\delta [I(t_{i+1})+R(t_{i+1})]+D(t_{i+1})\}-\{\delta [I(t_i)+R(t_i)]+D(t_i)\}\\ {{\tilde{D}}}_i&= D(t_{i+1})-D(t_i) \end{aligned}$$

Hence we compare the data *X* to the model output ${{\tilde{I}}}$ and $X^{(j)}$ to ${{\tilde{I}}}^{(j)}$, as well as *Z* with ${{\tilde{D}}}$ and $Z^{(j)}$ with ${{\tilde{D}}}^{(j)}$. At time $t_i$, our model validation is subject to measurement error, which is assumed to be of degenerate multivariate Gaussian distribution with mean $(X_i,Z_i)$ or $(X^j_i,Z^j_i)$ and covariance matrix $\Sigma$ or $\Sigma ^j$, where one covariate corresponds to the measurement error from confirmed cases and the other to the deceased cases. The time invariance of the covariance matrix was opted only for the sake of simplicity. Further simplification may assert prior assumption that the covariance terms in the measurement error are zero, meaning that each error is an independent process. This leads us to $\Sigma = \text {diag}(\sigma _Y,\sigma _Z)$ or $\Sigma ^j = \text {diag}(\sigma ^j_X,\sigma ^j_Z)$. Our likelihood function for only time point $t_i$ reads as

14 $$\begin{aligned} L_i(u):=\frac{1}{2\pi \sigma _X\sigma _Z}\exp \left( -\frac{({{\tilde{I}}}_i-X_i)^2}{\sigma _X^2}-\frac{({{\tilde{D}}}_i-Z_i)^2}{\sigma _Z^2}\right) . \end{aligned}$$

Assuming iid processes for all measurements at all time points, Kalbfleisch [28] pointed out a constant $K=(2\pi )^N$ that serves to simplify the joint likelihood function

15 $$\begin{aligned} L(u) &=K\prod _i L_i(u) \\ &= \frac{1}{\sigma _X^N\sigma _Z^N}\exp \left( -\sum _i\frac{({{\tilde{I}}}_i-X_i)^2}{\sigma _X^2}+\frac{({{\tilde{D}}}_i-Z_i)^2}{\sigma _Z^2}\right) . \end{aligned}$$

Our study designates the standard deviations as to approximate the means of confirmed and deceased cases, $\sigma _Y:=\Vert X\Vert /N$ and $\sigma _Z:=\Vert Z\Vert /N$. Defining *J*(*u*) as the sum of squares error of the difference between data and estimation using the parameter set *u*, i.e.,

16 $$\begin{aligned} J(u)&= \sum _{i}\frac{({{\tilde{I}}}_i-X_i)^2}{\Vert X\Vert ^2}+\frac{({{\tilde{D}}}_i-Z_i)^2}{\Vert Z\Vert ^2}, \end{aligned}$$

the likelihood and log-likelihood function then read as

17 $$\begin{aligned} L(u)&= \frac{N^{2N}}{\Vert X\Vert ^N\Vert Z\Vert ^N}\exp \left( -N^2 J(u)\right) , \end{aligned}$$

18 $$\begin{aligned} \log L(u)&= \log \left( \frac{N^{2N}}{\Vert X\Vert ^N\Vert Z\Vert ^N}\right) -N^2 J(u). \\ &=N \left[2\log N- \log \Vert X\Vert -\log \Vert Z\Vert -NJ(u)\right]. \end{aligned}$$

As the calculation can be done equivalently for the destination countries (*j*), the log-likelihood $\log L^{(j)}(u)$ is defined as

19 $$\begin{aligned} \log L^{(j)}(u)= N^{(j)} &\left[ 2\log N^{(j)} - \log \Vert X^{(j)}\Vert \right. \\ & \left. -\log \Vert Z^{(j)}\Vert -N^{(j)}J^{(j)}(u) \right]. \end{aligned}$$

### Model specification

The aim in model specification for the fitting of the data is that we have a measure (criterion) based on fit and complexity (information-type criterion). Therefore, regarding models A, B, and C, we opt for a minimal value of the Bayesian Information Criterion

20 $$\begin{aligned} \text {BIC}=\log N \cdot |u|-2\log L(u) \end{aligned}$$

according to Raftery [29], whose first term measures complexity represented by the observation size *N* and the number of parameters |*u*|, while the second term represents the maximal likelihood function. Note that for the travel destination countries, we do not compare the model output as we only allow the travel-independent system (4). The BIC penalizes the number of parameters more than the Akaike Information Criterion (AIC) [30], where the latter would have replaced the factor $\log (N)$ by 2. As far as model specification is concerned, our aim will be to choose between three models by selecting the model with minimal BIC as well as amending the question if the role of travellers is significant.

### Metropolis algorithm

In our study, we use a Metropolis algorithm (cf. Metropolis et al. [31], Gelman et al. [32] or Gilks et al. [33]) for estimation of parameters in the ODE systems (1) and (4) according to the procedure described in Schäfer and Götz [34] and Heidrich, Schäfer et al. [17]. Using the parameter set $u_0$ as of Table 4 as starting conditions, we assign random draws $u_{new}$ from a normally distributed (and thus symmetric) proposal function *q*, i.e. $u_{new} \sim q(u_{new}|u_{i-1})$, in every iteration *i*.

**Table 4:** Orders of magnitude of the initial values for adapting the model to the available data

Param.	$\beta _{0/1}$	$\delta$	$\mu$	$\tau$	$\alpha _j$	$E_0$
Init. val.	0.1	0.3	0.005	20	1	$1/2\cdot I_0$

Using the previously defined *J*(*u*) as the target distribution, we calculate the approximative distribution by

21 $$\begin{aligned} \pi (u)=c \cdot \exp {\left( -{\frac{J(u)^2}{2 \sigma ^2}}\right) }, \end{aligned}$$

whereby *c* is an arbitrary real value. For the acceptance probability, it follows

22 $$\begin{aligned} p(u_{new}|u_{i-1})&=\min \left\{ 1, \frac{\pi (u_{new})\cdot q(u_{i-1}|u_i)}{\pi (u_{i})\cdot q(u_{i}|u_{i-1}))}\right\} \\ &=\min \left\{ 1, \frac{\pi (u_{new})}{\pi (u_{i})}\right\} . \end{aligned}$$

In Eq. (22) we can see that the value of *c* is redundant as it cancels out in the division. If the sample is accepted with the probability *p*, we set $u_i=u_{new}$; with the probability $1-p$, the sample is declined, meaning $u=u_{i-1}$ according to Rusatsi [35] or Schäfer and Götz [34].

### Confidence intervals of the parameters

Considering that the observation size *N* and the number of parameters |*u*| hold the relation $N\gg |u|$, we adopt the idea of asymptotic confidence interval proposed in Teukolsky et al. [36]. Together with Raue et al. [37], these authors suggest that the asymptotic confidence interval can be a good approximation of the uncertainty in the optimal parameters $u^*$ providing that, besides the aforementioned relation, the measurement error is relatively small as compared to the data. The formula of the confidence interval for each parameter $u^*_k$ is given by $\text {CI}_k:=\left[ u_k^*-\psi , u_k^*+\psi \right]$, with $\psi$ being defined as

23 $$\begin{aligned} \psi :=\sqrt{2\chi ^2(q,df)\cdot \left( \nabla ^{-2}(-\log L(u^*))\right) _{kk}}. \end{aligned}$$

The operator $\nabla ^{-2}$ denotes the inverse of the Hessian while $\chi ^2(q,df)$ denotes the *q* quantile of the $\chi ^2$ distribution with the degree of freedom *df*. The degree of freedom can be chosen between two that further determines the type of confidence interval: $df=1$ gives the *pointwise asymptotic confidence interval* (PACI) that works on the individual parameter, $df=|u|$ gives the *simultaneous asymptotic confidence interval* (SACI) that works jointly for all the parameters [36].

### Current reproductive number

We also calculated the current 7-day reproduction number as of Götz et al. [38]: Defining the reproduction number ${\mathscr {R}}_{7,t}$ as the 7-day moving average of the infection cases at time *t* to the infection cases at time $t-3$ (assuming an incubation period of $\kappa ^{-1}=3$ days), we have

24 $$\begin{aligned} {\mathscr {R}}_{7,t}=\frac{\sum _{k=0}^{6} I_{t-k}}{ \sum _{k=0}^{6} I_{t-3-k}}. \end{aligned}$$

This ratio will be helpful to compare the results to the given infection data and find estimates on how the disease dynamics behave at least shortly after the investigated time interval.

### Sensitivity analysis

To answer questions (Q1) and (Q2), the basic idea of sensitivity analysis lies in the definition of a certain measure ${\mathscr {M}}$ for variable change that is worth of investigation, especially when one would like to describe its sensitivity with respect to a parameter $\vartheta$. The sensitivity of ${\mathscr {M}}$ with respect to $\vartheta$ in the sense of first-order change can be measured using Taylor expansion. Suppose that $\vartheta$ is increased to a certain percentage $\varepsilon$ from its current value, i.e., $\vartheta \mapsto \vartheta +\varepsilon \vartheta$. This way, the ratio $(\vartheta +\varepsilon \vartheta )/\vartheta =1+\varepsilon$ returns the total percentage post perturbation and $\varepsilon$ denotes the additional percentage of gain. Note that imposing $\varepsilon$ as the percentage is considered more robust than as simply the increase, considering that different parameters may live in disparate scales. Now, in the similar manner as for the parameter, the total percentage in ${\mathscr {M}}$ post perturbation on $\vartheta$ is given by

25 $$\begin{aligned} \frac{{\mathscr {M}}(\vartheta +\varepsilon \vartheta )}{{\mathscr {M}}(\vartheta )}=1+\varepsilon \vartheta \frac{\partial _\vartheta {\mathscr {M}}(\vartheta )}{{\mathscr {M}}(\vartheta )}+{\mathscr {O}}(\varepsilon ^2) \end{aligned}$$

providing that $\varepsilon$ is sufficiently small. Since the percentage of gain is usually considered similar across parameters, the role of $\varepsilon$ in the preceding equation is often neglected. The remaining expression thus provides a measurement of the sensitivity. Usually, authors refer $\partial _\vartheta {\mathscr {M}}(\vartheta )$ as the *sensitivity index* and $\vartheta \partial _\vartheta {\mathscr {M}}(\vartheta )/{\mathscr {M}}(\vartheta )$ as the *elasticity*, cf. Rockenfeller et al. [39]. Between two parameters $\vartheta _1,\vartheta _2$, it is logical to say that ${\mathscr {M}}$ is more sensitive to $\vartheta _1$ than $\vartheta _2$ when the absolute normalized sensitivity indices hold the relation

26 $$\begin{aligned} \left|\vartheta _1\frac{\partial _{\vartheta _1} {\mathscr {M}}(\vartheta _1)}{{\mathscr {M}}(\vartheta _1)}\right|> \left|\vartheta _2\frac{ \partial _{\vartheta _2} {\mathscr {M}}(\vartheta _2)}{ {\mathscr {M}}(\vartheta _2)}\right|. \end{aligned}$$

#### Time-dependent measures

The question (Q1) conveys the notion of model solution and addresses what our model solutions, including those excluded from the measurement or fitting, could have changed as we perturb the optimal parameter set, i.e. $\Lambda =\{\beta , \alpha , E_T ,\kappa ,\mu ,\gamma ,\tau \}$. Our interest is now driven by all the measures ${\mathscr {M}}$ that represent model state variables $\Psi =\{S,E,I,R,D\}$, which apparently are time-varying. To reveal the elasticity, one first compute the sensitivity index of state $\psi _i\in \Psi$ with respect to parameter $\lambda _j\in \Lambda$:

27 $$\begin{aligned} S_{ij}:=\frac{\text {d}}{\text {d} \lambda _j} \Psi _i \end{aligned}$$

from the sensitivity system of equations (cf. [39]):

28 $$\begin{aligned} S'_{ij}=\sum \limits _{k} S_{kj} \cdot \frac{\partial }{\partial \psi _k}f_i+\frac{\partial }{\partial \lambda _j}f_i, \qquad S_{ij}(0)=0 \;. \end{aligned}$$

The function *f* above defines the vector field of the model system, i.e., ${\dot{\Psi }}=f(t,\Psi ,\Lambda )$.

#### Time-independent measures

The question (Q2) is concerned more with interventions. In this case, we focus more on parameters that can be changed with the help of humans. In our context, such parameters could be $\beta$ and $\alpha$. The direct transmission rate $\beta$ has always been related to the proximity of the susceptible against infected humans and can be reduced with the aid of masks and social/physical distancing. The parameter $\alpha$ is related additional factors that drive the infection more than it could have been in the origin and destination country. For example, travellers are more exposed to physical encounters with other humans during flights, in public transportation, or in touristic areas, whereas locals spend more time at home. More protective apparatuses and educational campaigns will help reduce $\alpha$. In this regard, two different measures for the sensitivity can be considered. For the first choice, we may take, for example, ${\mathscr {M}}:=\int _0^TI\,\text {d}t$, which represents the total number of infected cases over all observations. If $\alpha ,\beta >0$, ${\mathscr {M}}$ is then more sensitive to $\beta$ rather than $\alpha$ when it holds

29 $$\begin{aligned} \beta \cdot \left|\frac{ \int _0^T\partial _{\beta }I\,\text {d}t}{ \int _0^TI \,\text {d}t}\right|> \alpha \cdot \left|\frac{\int _0^T\partial _{\alpha }I\,\text {d}t}{\int _0^T I\,\text {d}t} \right|. \end{aligned}$$

This inequality, however, includes the terms $|\int _0^T\partial _{\beta }I\,\text {d}t|,|\int _0^T\partial _{\alpha }I\,\text {d}t|$ that do not account for entropy or state of disorder. However, it is possible that the integral vanishes due to oscillations of the integrand $\partial _{\alpha }I$. This will result in a small sensitivity index rather than $\partial _{\beta }I$ that just forms a ‘calm’ trajectory above zero, so that the result would not be meaningful. To account for the entropy, we shall therefore consider the second measure

30 $$\begin{aligned} {\mathscr {M}}:=\int _0^{{{\hat{\beta }}}}\int _0^T|\partial _{\beta }I(t,s)|\,\text {d}t\text {d}s, \end{aligned}$$

which represents the total variation of *I* with respect to $\beta$, evaluated up to the current parameter value ${{\hat{\beta }}}$. Now, ${\mathscr {M}}$ is said to be more sensitive to $\beta$ than $\alpha$ (or vice versa) if

31 $$\begin{aligned} {\hat{\beta \cdot }} \left|\frac{ \int _0^T|\partial _{\beta }I|\,\text {d}t}{ \int _0^{{{\hat{\beta }}}}\int _0^T|\partial _{\beta }I(t,s)|\,\text {d}t\text {d}s}\right|>{\hat{\alpha }}\cdot \left|\frac{ \int _0^T|\partial _{\alpha }I|\,\text {d}t}{ \int _0^{{{\hat{\alpha }}}}\int _0^T|\partial _{\alpha }I(t,s)|\,\text {d}t\text {d}s}\right|. \end{aligned}$$

From the computational perspective, one can define a certain grid representing domain of interest for the two parameters, for example $[\beta _{\min },\beta _{\max }]\times [\alpha _{\min },\alpha _{\max }]$. The next step follows from computing the sensitivity indices for all grid points and applies the ratio of actual total variation and accumulated total variation as in Eq. (31). Therefore, the left-hand side should be done via stepping $\alpha$ (vertical mode) and the right-hand side via stepping $\beta$ (right mode).

## Numerical results

The number of iterations for Germany using the Metropolis algorithm, as well as for the preprocessing in each country should be a high number to prevent the algorithm from local minima. As in our previous work in Heidrich, Schäfer et al. [17] we set this number to 20,000. While the estimation was done for the daily cases, we plot the cumulated infection and dead because of better visibility. The reported cumulated cases consist of the currently infected cases plus the recovered plus the deceased cases, which are calculated as above by $\delta (I+R)+D$.

### Model A

**Fig. 2:**
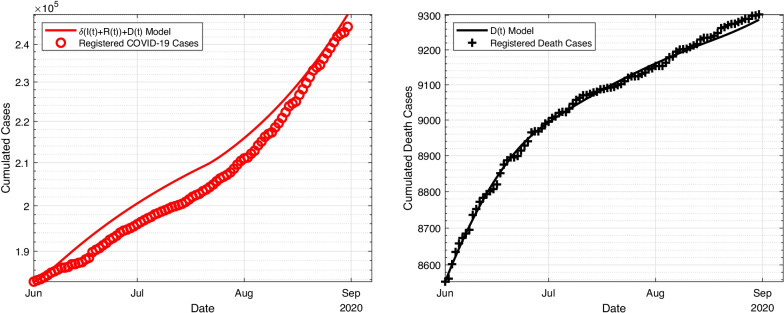
Estimation of cumulated infections in Germany compared to Johns Hopkins University from 1 June to 31 August with two piecewise constant values of the transmission rate $\beta$, on the left side the number of infections, on the right side the death cases. Note that, for better visibility, the y-axis does not include 0

To be able to compare the output of the optimal solutions for the three models, the result of the model with a piecewise constant value for $\beta$ and no traveller compartment is shown in Fig. 2. The optimization seems to be fairly decent for the death curve (right figure), but the model overestimates the infection cases (left figure) between June and August, which also shows in lower values for *L*(*u*) as seen in Table 8. In Table  5 the mean and standard deviations for the estimated parameters of the above explained model, starting values and methods are shown. Several parameter estimates are not very reliable, like the detection rate of $18\%$ which is expected to be higher due to comparably few cases yet an increased amount of available tests. For example, the study of Radon et al. [40] suggests a dark figure of slightly less than 50% in total until November. The death rate of 1% also appears to be much lower than expected (around 1%; for example, Dimpfl et al. [41] calculated a fatality rate of $0.83\%$ for the first wave, while Morwitzky et al. [42] found a fatality rate of $2.15\%$ as of November 2020). These findings suggest that Model A might not be a decent model to describe the disease behaviour in Germany in summer 2020.

**Table 5:** Numerical results for Model A without inclusion of $\alpha$

Parameter	Mean value	$\sigma$ of Metropolis
$\beta _0$	$8.65 \times 10^{-2}$d$^{-1}$	$0.19\times 10^{-2}$d$^{-1}$
$\beta _1$	$1.39\times 10^{-1}$d$^{-1}$	$0.01 \times 10^{-1}$d$^{-1}$
$\delta$	$1.84\times 10^{-1}$	$0.03 \times 10^{-1}$
$\mu$	$1.56\times 10^{-3}$	$0.06\times 10^{-3}$
$\tau$	$2.60 \times 10^1$d	$0.04\times 10^1$d
$E_0$	$3.60 \times 10^3$	$0.05\times 10^3$

### Model B

For Model B with a constant value for $\alpha$ from 15 June onwards, Table 6 shows the mean and standard deviations for the estimated parameters of the above explained model, starting values and methods. The estimated parameters, as far as known, are in line with what is to be expected. At the beginning of the investigated time interval, a rough estimate for the basic reproduction number without travellers is ${\mathscr {R}}_0=\beta /\gamma \approx 0.4$. As $\beta$ denotes the transmission rate at the beginning of June, without any effect of travellers, this estimate seems to be valid, but less than expected. A detection rate of $50-60\%$ as well as a death rate of $0.6\%$ are also valid estimates at the observed time interval. The time lag between infection and death is obviously dependent on the day of the registration of both infection and death, where 4 weeks is a decent approximation as well. Additionally, the pointwise asymptotic confidence interval and simultaneous asymptotic confidence interval are shown by $\psi$ as of Eq. (23), so that the respective interval is defined as $\text {CI}_k:=\left[ u_k^*-\psi , u_k^*+\psi \right]$.

**Fig. 3:**
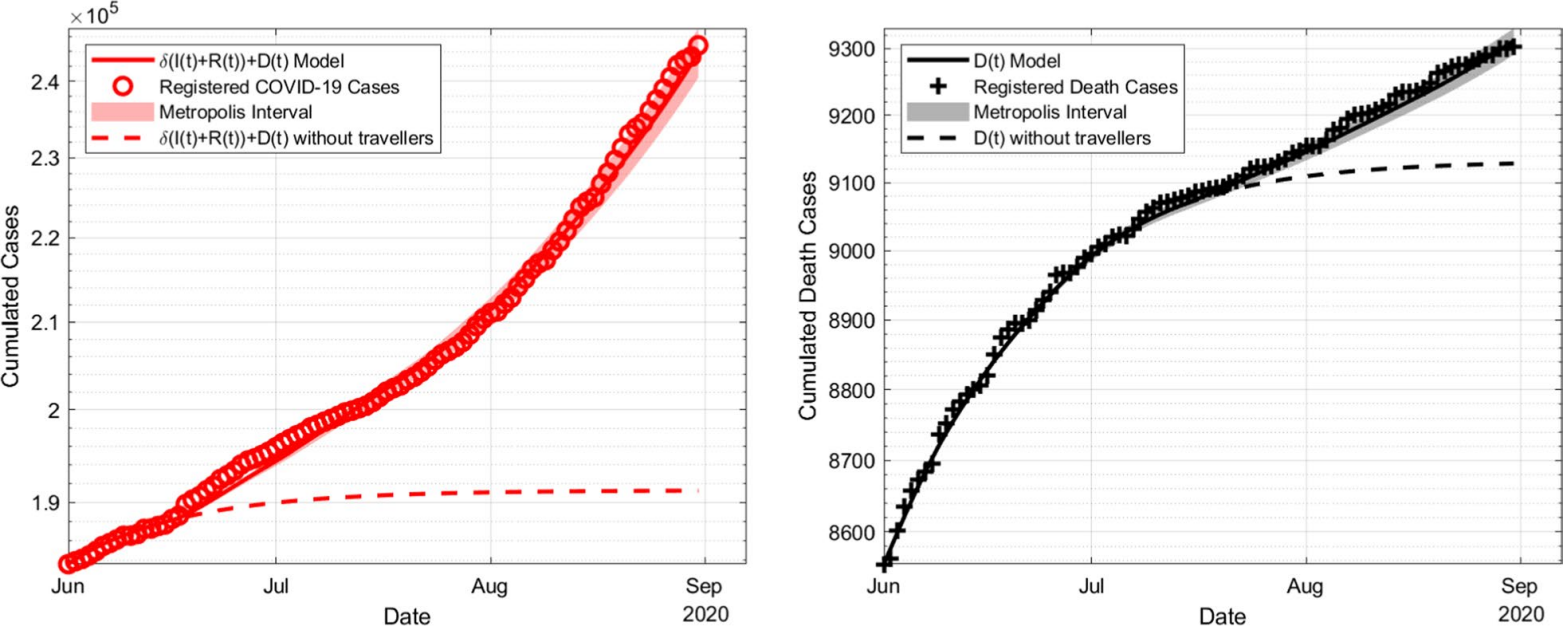
Estimation of cumulated infections in Germany compared to Johns Hopkins University from 1 June to 31 August with a constant impact rate $\alpha$, on the left side the number of infections, on the right side the death cases. Note that, for better visibility, the y-axis does not include 0. The (barely visible) shaded area represents the range of the solutions from the SACI and the dashed line describes the simulation with $\alpha =0$, i.e. either no travelling is allowed or the traveller compartment had been completely free of the disease

Figure 3 shows the estimated disease dynamics in comparison to the registered cases using the parameters as of Table 6. Additionally, the uncertainty range raised by the confidence intervals of the Metropolis algorithm is provided. For this, we add or subtract the standard deviation to or from the mean of the parameter to show the highest or lowest possible values of the registered cumulative infected persons. The range of both PACI and SACI is comparatively lower and almost no differences could be detected in the graphic. It is also observed how large the infected cases and fatalities in this model had been if $\alpha =0$, i.e. the travel ban had *not* ended and travellers had no impact on the disease dynamics whatsoever.

The left graphic in Fig. 3 shows that for our estimated parameter set, around 50,000 less infections with COVID-19 had been registered if the travel compartment had not been active. In the right figure concerning the death cases would make significant changes only from the end of July, resulting in a difference of roughly 150 death cases. As both the number of infected and the infection rates are higher than in the simulation with no traveller numbers, travellers appear to have created increased infection numbers at least at the beginning of September. However, a reasonable prediction on case numbers appears to be difficult, as interventions by authorities and the public (higher awareness due to higher infection numbers) cannot be predicted in the sense of an pre-calculable change of transmission rates.

**Table 6:** Numerical Results for Model B using a constant value of $\alpha$

Parameter	Mean value	$\sigma$ of Metropolis	$\psi$ of PACI	$\psi$ of SACI
$\beta$	$3.59 \times 10^{-2}$d$^{-1}$	$0.03\times 10^{-2}$d$^{-1}$	$0.002\times 10^{-2}$d$^{-1}$	$0.04\times 10^{-2}$d$^{-1}$
$\delta$	$5.78\times 10^{-1}$	$0.18 \times 10^{-1}$	$0.002 \times 10^{-1}$	$0.03 \times 10^{-1}$
$\mu$	$6.18\times 10^{-3}$	$0.19\times 10^{-3}$	$0.004\times 10^{-3}$	$0.09\times 10^{-3}$
$\tau$	$2.59 \times 10^1$d	$0.04\times 10^1$d	$0.001\times 10^1$d	$0.03\times 10^1$d
$E_0$	$2.59 \times 10^3$	$0.05\times 10^3$	$0.001\times 10^3$	$0.03\times 10^3$
$\alpha$	2.97	0.06	0.002	0.04

### Model C

**Fig. 4:**
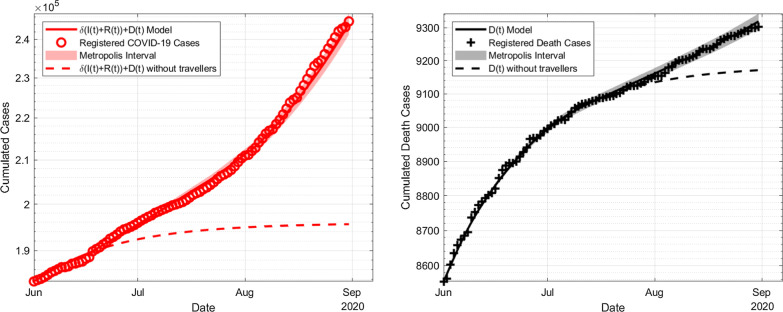
Estimation of cumulated infections in Germany compared to Johns Hopkins University from 1 June to 31 August with three piecewise constant travel impact rates $\alpha$, on the left side the number of infections, on the right side the death cases. The shaded area represents the SACI interval. The dashed line describes the simulation with $\alpha =0$, i.e. either no travelling is allowed or the traveller compartment had been completely free of the disease

For Model C, we now assume that $\alpha$ is not constant over the whole time from June to August, but rather time-dependent, defining a piecewise constant function of $\alpha$ with three different values. With $\alpha (t)$ being piecewise constant for 15–30 June, July and August, the parameter estimation for system (4) yields the following results as to be seen in Table 7. Parameter estimates are similar to those of Model B by the order of magnitude and thus equally reliable. In Fig. 4, similar to above, we show estimates and measured data for the cumulated cases and also the error range with respect to the Metropolis algorithm (which is the largest deviation) and the scenario if no travelling had been allowed.

**Table 7:** Numerical results for piecewise constant values of $\alpha$

Parameter	Mean value	$\sigma$ of Metropolis	$\psi$ of PACI	$\psi$ of SACI
$\beta$	$5.09 \times 10^{-2}$d$^{-1}$	$0.12\times 10^{-2}$d$^{-1}$	$0.002\times 10^{-2}$d$^{-1}$	$0.06\times 10^{-2}$d$^{-1}$
$\delta$	$4.94 \times 10^{-1}$	$0.08 \times 10^{-1}$	$0.007 \times 10^{-1}$	$0.19\times 10^{-1}$
$\mu$	$5.34 \times 10^{-3}$	$0.10\times 10^{-3}$	$0.004\times 10^{-3}$	$0.11\times 10^{-3}$
$\tau$	$2.58 \times 10^{1}$d	$0.06 \times 10^{1}$ d	$0.01 \times 10^{1}$ d	$0.34 \times 10^{1}$ d
$E_0$	$2.45 \times 10^3$	$0.03\times 10^3$	$0.003\times 10^3$	$0.07\times 10^3$
$\alpha _0$	2.23	0.05	0.002	0.06
$\alpha _1$	2.45	0.05	0.008	0.02
$\alpha _2$	3.14	0.05	0.004	0.11

### Comparison

For the Bayesian analysis, we can now compare the BIC values of the three models computed by Eqs. (18) and (20). The results for Model A were gained by applying Eq. (4), i.e. the model we used to estimate the disease behaviour in all other countries (with no travel impact rate, but two piecewise constant transmission rates $\beta _{1,2}$) to Germany.

Table 8 shows that in terms of the least-square output, the model with time-dependent, piecewise constant values of $\alpha$ (Model C) shows the best results. Even though the penalization of complexity with two more parameters, the BIC for model C is the lowest. According to Raftery [29], a BIC difference of 6–10 indicates a “strong” evidence (posterior probability of 95–99%) that Model C using three piecewise constant values for $\alpha$ is to be preferred over B, while there is “very strong” evidence that model C, and also B, are to be preferred over model A with a posterior probability of $>99 \%$ as the difference is larger than 10.

**Table 8:** Values for the least-square value *J*(*u*) and the BIC for the various models

	*J*(*u*)	# of Parameters	BIC
Model A	$4.4174 \times 10^{-5}$	6	− 9087.0
Model B	$4.1812 \times 10^{-1}$	6	− 8538.6
Model C	$4.1919 \times 10^{-1}$	8	− 8529.9

**Fig. 5:**
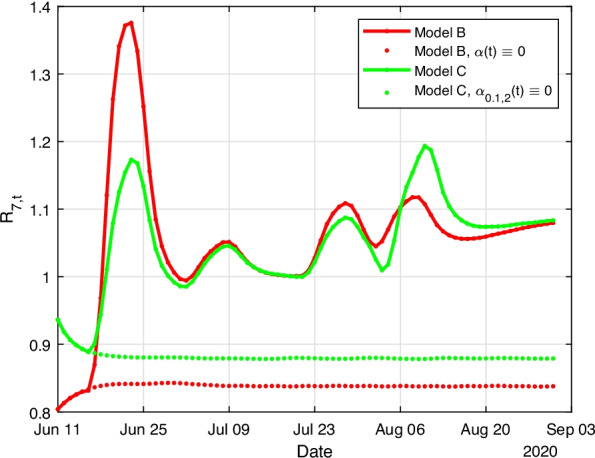
Reproductive values $R_{7,t}$ for the measured infection values, Model B (red) and Model C (green). Additionally, the dotted lines show the evolution of the reproductive values in case travellers had no impact on the infections in Germany

Lastly, we compare the 7-day reproductive number as of Eq. (24). Figure 5 shows the values for the two models. Both curves have a similar behaviour and the values of $R_{7,t}$ in Model B and C would remain $\gtrapprox 1$ most of the time, resulting in growing infected values even for at least a short time after the investigated time window, i.e. at the beginning of September.

Additionally, we plotted the dynamics of $R_{7,t}$ for the hypothetic case that no travellers had contributed to the infection cases. In this case the values of $R_{7,t}$ would remain $<1$ for the whole time in any of the two models. This means the disease would have been contained if no other effects are assumed.

We can compare future simulations on a short-time scale by extending the time interval of the model. When we assume that travelling had not been allowed during the whole time interval $\alpha (t)\equiv 0$, a simulation until September 15 assumes only 100 new registered infections. However, in the estimation of model C where travelling is allowed, we computed 21,200 new infections if travelling had been disallowed from September 1 onwards and 26,300 new infections if the conditions for travelling had not been changed at all between September 1 and 15 when we assume the situation is not changed by any national or international measures, i.e. same travel numbers and impact rates as at August 31 are assumed. Results for model B show similar values in terms of magnitude; it is important to note that values of those estimates are to be taken with caution.

### Sensitivity analysis

**Fig. 6:**
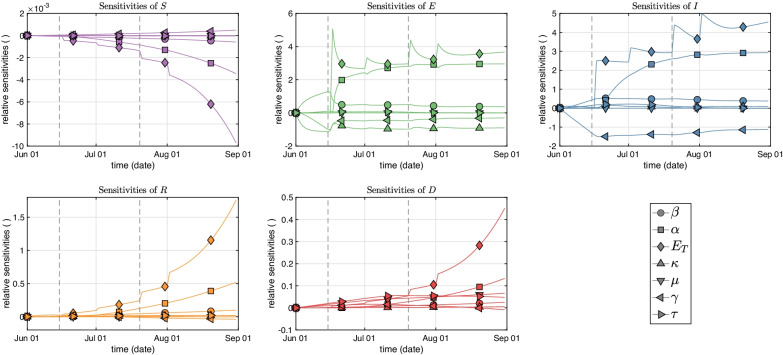
Sensitivities of the model states with respect to its parameters, using a time-independent travel impact rate

For the investigation of time-dependent measures, we included the parameters $\gamma$ and $\kappa$ from Table 1 although they were not optimized, yet can be assumed to bear uncertainties, to observe the influence of those parameters to the solutions for all five compartments *S*, *E*, *I*, *R*, and *D*. For Model B, all the parameters $\lambda _i$ are almost constant, except $E_T$ and $\alpha$, and to some extent $\beta$ and $\gamma$ (latter of which is however not a mutable parameter in our optimization). Figure 6 shows the elasticities $\lambda _jS_{ij}/\Psi _i$ using (27) around the parameter values given in Tables 6 and 7, respectively. Generally, $E_T$ returns the highest sensitivity in all compartments, particularly with ongoing simulation time. Additionally, changes in the parameters $\alpha$ (after a certain time delay, mainly due to differing from zero only after June 15) and also $\gamma$ would significantly influence the infected compartment but not the death compartment, in which no parameter shows a higher elasticity than 0.15. Note that the parameter $E_T$ is actually proportional to $\alpha$ as of Eq. (3). After all, a caveat with these measures remains, as the elasticities are time-varying. Therefore, preference to a certain parameter for the highest elasticity could change over time.

**Fig. 7:**
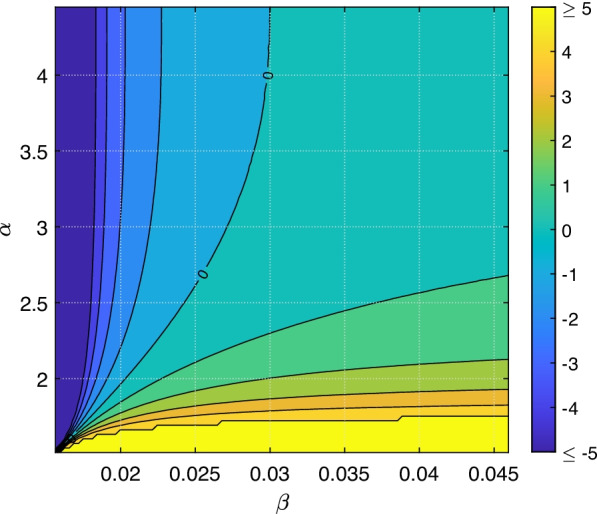
Comparison of the elasticities in a domain of interest for the transmission rate $\beta$ and the travel impact rate $\alpha$. The area shows the contour of the elasticity in the left-hand side of Eq. (31) minus the right-hand side expression. The region below the zero-line thus indicates all possible locations of $(\beta ,\alpha )$ at which the measure ${\mathscr {M}}$ as in Eq. (30) is more sensitive to $\beta$ than to $\alpha$, and vice versa

Concerning the time-independent measure, we can now generate a two-region profile for which the inequality in Eq. (31) indeed applies or the other direction does. On the basis of Model B for the portraying the upcoming winter outbreaks, Fig. 7 shows the comparison of the elasticities in reasonable ranges of $\beta$ and $\alpha$, the two parameters where interventions actually can change values. While reducing the overall transmissions in some way is equivalent to a reduction of $\beta$, reduction of travellers can be interpreted as a reduction of $\alpha$: Even if the value $\alpha$ is not related to the amount of travellers, we have seen that $\alpha$ is multiplied with the amount of travellers $T_{\text {Germany}\leftrightarrow j}$. If this value is reduced, then the product is reduced by the same factor, which would yield the same results as a reduction of $\alpha$ by this factor. Alternatively, travel control without reduction of traveller numbers can also reduce $\alpha$. Using the fitted value $(\alpha ,\, \beta )=(2.97,\, 0.0309)$ we find that the measure ${\mathscr {M}}$ as in Eq. (30) is more sensitive to $\beta$ than $\alpha$. This finding draws forth further practical relevance. Our model can be calibrated with new incidence data on an initial take-off period in the next winter season, where all parameters except $\beta$ and $\alpha$ are fixed according to our fitting. At first, the two parameters can be fitted to these new data. May they locate in one of the two regions separated by the zero-curve in Fig. 7, we then acquire knowledge on which resources should be drawn in order to attack the most sensitive parameter. One can thus wait and see how the deployment of the resources gives the real-time intervention to the number of infected cases. Re-calibration then follows after some time as short-term feedback from such an intervention is gained, and the values of optimal $\beta$ and $\alpha$ can once again be evaluated via Fig. 7. This process of combining sensitivity-based interventions remains continuous until the ultimate disease eradication is achieved without having to waste resources.

## Discussion and conclusion

In this present work we intended to measure the impact of travellers on the overall disease dynamics in Germany during summer 2020 using a modified SEIRD-model with a traveller compartment. Travel rates are measured by using international flight and hospitality data. The infection data of all 55 countries with more than 5000 German travellers in June, July and August together has been used to optimize the single-country infections. Estimates for the transmission rates $\beta _{j,0/1}$ and the infected $I_j$ at all time *t* in those countries are found using standard SIRUV models and used to estimate the travel impact rate for Germany. Parameter estimation was done using a Metropolis type algorithm, while other routines like an adjoint based approach are also possible.

The estimated parameter values of the travelling-induced models are generally close to medical estimations, while the model with time-dependent transmission rates delivers less reasonable results in terms of the target function and also the parameter values. In the traveller models, the travel impact rate was estimated to be in the range of $2.2 \le \alpha \le 3.2$, meaning a two-to three times higher infection possibility as a traveller than the average inhabitant of the respective country. The model with three time-dependent and piecewise constant values of the travel impact rate $\alpha (t)$ yields better values than a model with only one constant value for $\alpha (t)$ in the ${\mathscr {L}}_2$ norm and also better BIC values despite two more parameters being used, and can be classified as very strongly preferred.

The raised infection numbers and infection rates by travellers are also assumed to have caused higher infection numbers at least in the following weeks (autumn 2020), based on an analysis of the reproductive number at the end of the investigated time interval. Among other reasons such as seasonality and opening of schools after the summer holidays, these are assumed to have an impact on the second large infection wave in late 2020 [2, 6]. It needs to be clear that due to the lack of precise data, traveller values can be only estimated to a certain degree and some data sets with which the parameter estimates for the various countries are not necessarily reliable. We aimed to reproduce the infection risks for travellers in those countries. Because of the large number of countries, errors are therefore assumed to be evened out as the value of $E_T$ is a sum of the infections from all those countries. Also, using piecewise constant values for $\alpha$ (to some extent also $\beta _j$) with switching dates as of the first of each month is slightly arbitrary. While a steady function $\alpha (t)$ or optimization of the switching dates in some way can lead to better results, those are prone to overfitting. The lower BIC of the model with three different values for $\alpha$ indicates this did not happen for model C.

Further, we performed a sensitivity analysis for model B, i.e., a constant value of $\alpha$. In particular, it is found that the sensitivities for the travel impact rate $\alpha$ can be identified after a certain time delay (which is caused by the model definition). The parameters $\alpha$, $E_T$—which has the same behaviour as $\alpha$ due to the construction of the model-, $\beta$ and $\gamma$ are found to be the most relevant parameters. However, in the further analysis we constricted toward $\beta$ and $\alpha$ as those are the parameters regarding which political interventions are possible. For those two parameters, we designed a two-region profile for which the detected domains in which a reduction of $\alpha$ is more relevant for disease control in case the transmission rate $\beta$, especially when the infection cases are otherwise comparatively low and can be controlled. Finding those domains is similar to the findings of Hollingsworth et al. [12], as they claimed travel bans are only relevant in case of low values for *R*, which can be interpreted as a reduction of the transmission rate $\beta$. In case of higher infectivity rates like for, e.g., the latest mutants (Delta and, even more so, Omikron), which go along with a larger value of $\beta$, those assumptions might thus not hold true in the same way. However, it might be reasonable to consider travelling restrictions for a supposed variant (or other disease) with comparatively low transmission rates yet high mortality, Still, the raised infection rates at the end of summer 2021 are an indicator that higher/‘uncontrolled’ traveller numbers might have been a reason for another (at that time ‘fourth’) wave one year after the investigated time interval, which can be part of future investigations.

Not letting aside that installation of travel restrictions has multiple political, legal, social and economical problems (it is not to be forgotten these pose an encroachment into fundamental rights), as we thus conclude that setting up travel policies can be an epidemiologically reasonable policy component to contain disease numbers at least for short terms, which is in line to the findings of papers [7–14]. Rather than an exportation of cases as in Siegenfeld et al. [7] or Chinazzi et al. [8] to several countries or internally in China as in Zou et al. [9], we consider importation of cases from many different countries with varying infection rates. Thus, unlike [9], we make general statements of the effectiveness of travel restrictions due to a combination of all countries in the equation for $E_T(t)$. The analysis of the local reproduction number suggest that the values are fluctuating around 1, and travel measures have the potential to below 1, resulting in an extincting disease. Additionally, short-time simulations for the beginning of September 2020 show a difference of several thousands of infection cases between no (further) travel restrictions and full travel restrictions. These two findings indicate that travel measures should be imposed alongside other social measures for optimal disease control. It is up to further research to regard whether solely a travel ban or tightening of travel restrictions had just postponed the third infection wave to a later date. Even if this latter assumption holds true, there are possible advantages of delaying the disease: Similar to Epstein et al. [13], the findings show that delaying the epidemics can be achieved inter alia by travel restrictions. This time can then be used to prepare for the income of an infection wave, prevents overload the health care system all at once (which is also found by Leung et al. [10]), or postpones epidemics until vaccines are available so that the amount of severe disease courses is reduced. Travel restrictions to farther countries is comparatively ‘cheaper’ than to closer ones, where border control is required which induces further political and social problems. While restrictions to a (lower) amount of high-risk areas like several articles propose can be more effective, global measures can be more effective or ‘safer’ than targeted measures in an epidemiological/stochastic sense, due to the highly connected world we live in and possibly rapid changes in the disease dynamics in single countries. Awareness of the dynamics of the Corona waves in previous years and its reasons is important in upcoming years of the pandemic and for other fast-spreading diseases as well, and at the start of a pandemic or at least a single wave, a strategy combining local social measures with international measures, in particularly a (heavy) reduction of traveller numbers, should be considered together in terms of optimal control especially when risks cannot be foreseen. It is also to be noted that travel measures are undertaken in a graded way. Details on related regulations have changed from time to time, but certain entry restrictions have been upheld since the earlier rise of the pandemic. For example, all persons entering the country must provide a negative test result or, later, proof of immunity either by recovery or vaccination, then comply with post-arrival quarantines depending on the place of departure. However, it remains a question which of those two to consider primarily, and for that modelling scenarios like the ones presented (e.g. Fig.7) can be updated on the current situation .

Further work in this topic might also include the impact of foreign travellers in Germany and a international multi-patch/network model including travellers from and to all investigated regions or countries. Also, other types of models, e.g. stochastic delayed differential equations (SDDE) or agent-based systems, can be used to model disease dynamics and incorporation of travellers.

## Data Availability

We used several of the underlying MATLAB codes for multiple purposes and thus believe the open presentation of the codes would distract readers. However, the codes, datasets used, and/or analysed during the current study which are not already cited in the article (cf. [2, 5, 19–22]), are available from the corresponding author on reasonable request.
